# Plasma and urine neutrophil gelatinase-associated lipocalin in septic and nonseptic ICU patients

**DOI:** 10.1186/cc10959

**Published:** 2012-03-20

**Authors:** CS Vrettou, S Kokkoris, K Apostolou, M Parisi, E Haritatos, S Dimopoulos, S Nanas

**Affiliations:** 1 National and Kapodistrian University of Athens, Greece; 2 Evaggelismos Hospital, Athens, Greece

## Introduction

In this prospective cohort study we investigate how admission plasma and urine neutrophil gelatinase-associated lipocalin (pNGAL and uNGAL) levels are affected by the presence of sepsis in a general ICU population. These novel biomarkers are currently being evaluated for acute kidney injury (AKI) prediction. However, they are also increased in sepsis, which can be a confounding factor regarding their specificity for AKI [1,2].

## Methods

Ninety-six patients consecutively admitted to the ICU were included in the study. Exclusion criteria were chronic renal failure, AKI prior to ICU admission, brain death, pregnancy, age <18 years and predicted ICU stay less than 48 hours. Patients' demographic characteristics, APACHE II and SOFA score, existing comorbidities, primary reason for admission to intensive care, pNGAL, uNGAL, white cell count and C-reactive protein levels were recorded on admission, while the RIFLE score and sepsis status were recorded until day 7 post admission. The Mann-Whitney U test was used to compare pNGAL and uNGAL levels in septic and nonseptic patients.

## Results

Out of 96 patients included, 56 were male, 12 had AKI and 30 had sepsis on admission. The mean age was 55.5 ± 19.6 years, the mean APACHE II score was 14.8 ± 5.6 and the mean admission SOFA score was 6.6 ± 2.9. There were 43 medical admissions, 17 elective surgical, and 36 emergency surgical including trauma.

Both pNGAL and uNGAL were higher in patients with AKI on admission (*P *< 0.001). Their levels were also found to be higher in septic compared with nonseptic patients (septic pNGAL = 153.13 ± 144.86 vs. nonseptic pNGAL = 102.45 ± 95.65, *P *= 0.076; septic uNGAL = 306.66 ± 532.88 vs. nonseptic uNGAL = 123.41 ± 354.07, *P *= 0.002). When patients with AKI as well as patients who developed AKI within the first 7 days post admission were excluded from the analysis, higher uNGAL and pNGAL values in the group of septic patients were not significant at the level of 5%. The estimated sample size for significance 5% and power 80% is 74 for uNGAL (2,200 for pNGAL). Moreover pNGAL and uNGAL had a similar area under the ROC curve (0.773 and 0.779 respectively) for predicting AKI in septic patients.

## Conclusion

Both biomarkers are increased in the case of sepsis in our population. Septic AKI affecting uNGAL more than pNGAL could explain the smaller *P *value for uNGAL in the group of patients with sepsis.
